# Supplementary material for: Dynalign II: common secondary structure prediction for RNA homologs with domain insertions
Source: Nucleic Acids Res. 2014 Nov 21;42(22):13939–48. doi: 10.1093/nar/gku1172 (PMC4267632; doi:10.1093/nar/gku1172)
Supplement: SUPPLEMENTARY DATA [file supp_gku1172_nar-02021-z-2014-File012.zip › manual/Text/dynalign.html]

RNAstructure Command Line Help: dynalign


|  |  |  |
| --- | --- | --- |
|  | RNAstructure Command Line Help dynalign | - Contents - Index |
| dynalign is used to find the lowest free energy common secondary structures for two homologous sequences. It has two distinct executables, a serial program called dynalign and a parallelized program called dynalign-smp for use in shared memory environments. dynalign\_ii supercedes dynalign and should be used fo rmost applications. dynalign\_ii allows inserts of structural domains that appear in only one of the two homologs. dynalign\_ii also allows unpaired nucleotides to align to paired nucleotides. dynalign\_ii-smp is a parallelized version for shared memory environments. Note that on Macintosh OS X, and some versions of Linux, there is a small default stack limit. To run Dynalign/Dynalign II, the stack limit needs to be increased. On the default shell, bash, use *ulimit -s 4096*. If you are using tcsh, use *limit stack 4096*. The limits can be increased as necessary if the given values above are not sufficient. USAGE 1: dynalign\_ii <configuration file> USAGE 2: dynalign\_ii-smp <configuration file> USAGE 1: dynalign <configuration file> USAGE 2: dynalign-smp <configuration file> Required parameters: <configuration file> \| The name of a file containing required configuration data. Options which do not require added values: NONE Options which require added values: NONE dynalign\_ii configuration file format: The following is a description of valid options allowed in the configuration file. The example is based on dynalign\_ii\_sample.conf, a standard example found in the examples directory of the RNAstructure repository. ``` ################################################################ # IMPORTANT CONFIG FILE FORMAT NOTES: # # Config file options described below are not case sensitive. # # Option lines may be specified by the option name followed by an equals sign and the option's desired value. # When specifying an option, there may be nothing else on the line. # If an option is specified more than once, the last specification is used. # <option> = <value> # # Specifying comment lines: # Comment lines must begin with "#" followed by a space. # There may not be more than one "#" in a comment line. # However, a comment line may be an unbroken string of "#", as in a divider between sets of options. # # Blank lines are skipped. # Any leading or trailing whitespace is ignored. # Variables may not contain internal whitespace. # # Syntax errors produce a warning to standard output and are then ignored. ################################################################ ################################################################ # Required input # If one of these values is not defined, the program will exit. ################################################################ #These are required input: inseq1 = RD0260.seq inseq2 = RD0500.seq outct = 1.ct outct2 = 2.ct aout = ali.ali ################################################################ # Options with default values if not explicitly specified # (Default values are shown) ################################################################# #fgap is the per nucleotide insert penalty for alignments: fgap = .4 #slope is the per nucleotide free energy penalty for inserted domains slope = 0.1 #intercept is the initiation free energy penalty for inserted domains intercept = 0.5 #maxtrace is the masximum number of predicted structures: maxtrace = 750 #percent is the maximum % change in free energy from the lowest free energy structure percent = 20 #bpwin is the base pair window bpwin = 2 #awin is the alignment window awin = 1 #singlefold_subopt_percent is the maximum % difference in folding free energy change #from single sequence folding for pairs that will be allowed in a subsequent Dynalign calculation. #This is used to save calculation time by pre-screening allowed pairs. singlefold_subopt_percent = 30 #imaxseparation is the traditional M parameter: #-99 indicates that the alignment constraint (preferred method is used) imaxseparation = -99 #maxelongation is the maximum length of a consecutive set of base pairs aligned with an internal loop with the same length maxelongation = 5 #num_processor is required only for smp (parallel) calculations num_processors = 1 #optimal only is optional, only the lowest free energy structure is calculated if optimal_only = 1 optimal_only = 0 #local alignment is performed if local = 1, the default is 0 (global alignment) local = 0 #The following are needed for progressive calculations #dsv_templated is set to 1 to read the template from previous calculation dsv_templated = 0 # dsvtemplatename = RD0260.RD0500.dsv #The following are used to predict a structure for sequence 2, where the structure for sequence 1 is known. #If ct_templated is set to 1, inseq1 must refer to a ct file, NOT a sequence file. ct_templated = 0 #The following parameters are used when SHAPE data is utilized (see below). #There is a set of parameters for each sequence. #shapeslope1 = 1.8 #shapeintercept1 = -0.6 #shapeslope2 = 1.8 #shapeintercept2 = -0.6 #The following can be used to run Dynalign using DNA thermodynamics instead of RNA. #Use DNA = 1 to do DNA structure prediction. DNA = 0 #The following is used to change the temperature from the default of 310.15 K (37 degrees C). temperature = 310.15 ################################################################ # Options that are not required and have no default values ################################################################ #savefiles are optional and are needed for dot plots # savefile = RD0260.RD0500.dsv #Folding constraints can be input using constraint files: #constraint_1_file = constraints_for_sequence1 #constraint_2_file = constraints_for_sequence2 #SHAPE data can be input using .shape files for either, neither, or both #	SHAPE is utilized using the pseudo free energy method of Deigan et al. #	PNAS 106:97 #shape_1_file = shape_for_sequence1 #shape_2_file = shape_for_sequence2 #Use constraint_align_file to enforce specific nucleotide alignments #constraint_align_file = aln.txt #Use maximumpairingdistance to limit the maximum distance between #	paired nucleotides (where the final # indicates the sequence #). #	Note that this only works for sequence 1 if the calculation is not #	cttemplated or dsvtemplated. #maximumpairingdistance1 = 600 #maximumpairingdistance2 = 600 ``` dynalign\_ii configuration file format: The following is a description of valid options allowed in the configuration file. The example is based on dynalign\_sample.conf, a standard example found in the examples directory of the RNAstructure repository. ``` ################################################################ # IMPORTANT CONFIG FILE FORMAT NOTES: # # Config file options described below are not case sensitive. # # Option lines may be specified by the option name followed by an equals sign and the option's desired value. # When specifying an option, there may be nothing else on the line. # If an option is specified more than once, the last specification is used. # <option> = <value> # # Specifying comment lines: # Comment lines must begin with "#" followed by a space. # There may not be more than one "#" in a comment line. # However, a comment line may be an unbroken string of "#", as in a divider between sets of options. # # Blank lines are skipped. # Any leading or trailing whitespace is ignored. # Variables may not contain internal whitespace. # # Syntax errors produce a warning to standard output and are then ignored. ################################################################ ################################################################ # Required input # If one of these values is not defined, the program will exit. ################################################################ inseq1 = <seq file 1> inseq2 = <seq file 2> outct = <output ct file for seq 1> outct2 = <output ct file for seq 2> aout = <output alignment file> ################################################################ # Options with default values if not explicitly specified # (Default values are shown) ################################################################# # fgap is the per nucleotide insert penalty for alignments: fgap = .4 # maxtrace is the masximum number of predicted structures: maxtrace = 750 # percent is the maximum % change in free energy from the lowest free energy structure: percent = 20 # bpwin is the base pair window: bpwin = 2 # awin is the alignment window: awin = 1 # insert indicates whether single basepair inserts will be allowed: insert = 1 # singlefold_subopt_percent is the maximum % difference in folding free energy change # from single sequence folding for pairs that will be allowed in a subsequent Dynalign calculation. # This is used to save calculation time by pre-screening allowed pairs. singlefold_subopt_percent = 30 # imaxseparation is the traditional M parameter: # -99 indicates that the alignment constraint (preferred method is used) imaxseparation = -99 # num_processor is required only for smp (parallel) calculations num_processors = 1 # optimal only is optional, only the lowest free energy structure is calculated if optimal_only = 1 optimal_only = 0 # local alignment is performed if local = 1, the default is 0 (global alignment) local = 0 # the following are needed for progressive calculations # dsv_templated is set to 1 to read the template from previous calculation dsv_templated = 0 dsvtemplatename = <template file name> # The following are used to predict a structure for sequence 2, where the structure for sequence 1 is known. # If ct_templated is set to 1, inseq1 must refer to a ct file, NOT a sequence file. ct_templated = 0 # The following parameters are used when SHAPE data is utilized (see below). # There is a set of parameters for each sequence. shapeslope1 = 1.8 shapeintercept1 = -0.6 shapeslope2 = 1.8 shapeintercept2 = -0.6 # The following can be used to run Dynalign using DNA thermodynamics instead of RNA. # Use DNA = 1 to do DNA structure prediction. DNA = 0 # The following is used to change the temperature from the default of 310.15 K (37 degrees C). temperature = 310.15 ################################################################ # Options that are not required and have no default values ################################################################ # Savefiles are optional and are needed for dot plots. savefile = <save file name> # Folding constraints can be input using constraint files: constraint_1_file = <constraint file for seq 1> constraint_2_file = <constraint file for seq 2> # SHAPE data can be input using .shape files for either, neither, or both # SHAPE is utilized using the pseudo free energy method of Deigan et al. # PNAS 106:97 shape_1_file = <SHAPE file for seq 1> shape_2_file = <SHAPE file for seq 2> # Use constraint_align_file to enforce specific nucleotide alignments. constraint_align_file = <alignment constraints file> # Use maximumpairingdistance to limit the maximum distance between # paired nucleotides (where the final # indicates the sequence #). # Note that this only works for sequence 1 if the calculation is not # cttemplated or dsvtemplated. maximumpairingdistance1 = <value for seq 1> maximumpairingdistance2 = <value for seq 2> 						 ``` Comparison of configuration file format: dynalign\_ii had added parameters slope, intercept, and max\_elongation, which apply only to dynalign\_ii calculations. dynalign has a parameter, insert, which no longer applies to dynalign\_ii. References: 1. Reuter, J.S. and Mathews, D.H. "RNAstructure: software for RNA secondary structure prediction and analysis." *BMC Bioinformatics*, 11:129. (2010). 2. Fu, Y., Sharma, G., and Mathews, D. H. "Dynalign II: Common Secondary Structure Prediction for RNA Homologs with Domain Insertions." In preparation. 3. Harmanci, A.O., Sharma, G. and Mathews, D.H. "Efficient Pairwise RNA Structure Prediction Using Probabilistic Alignment Constraints in Dynalign." *BMC Bioinformatics*, 8:130. (2007). 4. Uzilov, A.V., Keegan, J.M. and Mathews, D.H. "Detection of non-coding RNAs on the basis of predicted secondary structure formation free energy change." *BMC Bioinformatics*, 7:173. (2006). 5. Mathews, D.H. "Using the RNAstructure Software Package to Predict Conserved RNA Structures." In Baxevanis, A. D., Davison, D. B., Page, R. D. M., Petsko, G. A., Stein, L. D. and Stormo, G. D. (eds.). *Current Protocols in Bioinformatics*. John Wiley and Sons, Inc., New York, pp. 12.14.1-12.14.22. (2014). 6. Mathews, D.H. "Predicting a set of minimal free energy RNA secondary structures common to two sequences." *Bioinformatics*, 21:2246-2253. (2005). 7. Mathews, D.H. and Turner, D.H. "Dynalign: An algorithm for finding the secondary structure common to two RNA sequences." *J. Mol. Biol.*, 317:191-203. (2002). | | |
| Visit The Mathews Lab RNAstructure Page for updates and latest information. | | |
